# Japanese gastric cancer treatment guidelines 2018 (5th edition)

**DOI:** 10.1007/s10120-020-01042-y

**Published:** 2020-02-14

**Authors:** Japanese Gastric Cancer Association

**Affiliations:** Japanese Gastric Cancer Association, 465 Kajii-cho, Kawaramachihirokoji, Kamigyo-ku, Kyoto, 602-0841 Japan

## Preface to the English version

This English version was made based on the Japanese version of the Japanese Gastric Cancer Treatment Guidelines published as a book in 2018. However, this version reflects some of the new evidence that emerged since the publication of the Japanese version.

## Preface to the Japanese Gastric Cancer Treatment Guidelines 5th edition

The 5th edition of the Japanese Gastric Cancer Treatment Guidelines was completed in January 2018, incorporating new evidence that emerged after publication of the previous edition. Information on some of the new evidence had already been delivered as quick bulletins in the website of the Japanese Gastric Cancer Association (JGCA) to supplement the previous edition.

Prior to the initiation of editing process, concept and style of the new edition were reconsidered by the committee members. A survey participated by the entire member of the JGCA regarding the style of the treatment guidelines revealed that the members preferred the conventional textbook style as a format of the new treatment guidelines. However, the current trend of guideline editing as observed in guidelines for other types of cancer is strongly influenced by the Medical Information Network Distribution Service (MINDS) which has established a clear definition of the guidelines, and created and publicized standard methodology for their compilation. Thus, the committee members created several relevant Clinical Questions (CQ) and attempted to provide the best possible answers accompanied with detailed explanations, including the levels of evidence and the strength of recommendations. Besides adhering to the philosophy of our senior members who compiled the first edition which was the first of the cancer guidelines issued in Japan, the current committee members made attempts to incorporate the methodology established by the MINDS. Consequently, there might be more drastic changes in the structure and style in the upcoming revision.

After publication of the previous edition in 2014, several pivotal randomized studies on surgery along with a prospective confirmative study focused on the endoscopic resection have been published. In the field of chemotherapy, emergence of new anti-cancer agents led to impressive increase in the options for therapeutic regimens. Therefore, considerable amount of revision was needed to compile the current version.

Major points of revision in the current edition are listed below:

1. Staging system of gastric cancer has been connected with recently issued Japanese Classification of Gastric Carcinoma 15th edition [1], and Union for International Cancer Control (UICC) TNM classification 8th edition [2].
2. The algorithm showing standard clinical practices was revised and each item that underwent a major change or remains to have an unsolved issue was linked to the corresponding clinical question.
3. The splenic hilar lymph node (No. 10) has been deleted from the definition of D2 lymph node dissection in total gastrectomy. Furthermore, results of other recent clinical studies in surgery have been reflected in the text.
4. The indication for endoscopic resection has been revised. A new classification of curability after endoscopic resection, termed “eCura” classification, was established based on the protest that the prefixes such as “non-curative” that had been used in the previous classification were inappropriate.
5. Chemotherapeutic regimens to treat unresectable advanced or recurrent gastric cancer were classified into either the “recommended regimen” or “conditionally recommended regimen”. The evidence level as defined in the MINDS manual ver. 2 was provided for all regimens that fall into the “recommended regimens” category.
6. Several important clinical questions in the fields of surgery, endoscopic resection and chemotherapy which would frequently be asked during the clinical practice were extracted by the committee members, and the corresponding recommendations and comments were provided.

## Treatments

### Treatment modalities and their indications

#### Algorithm of standard treatments to be recommended in clinical practice

**Fig. 1:**
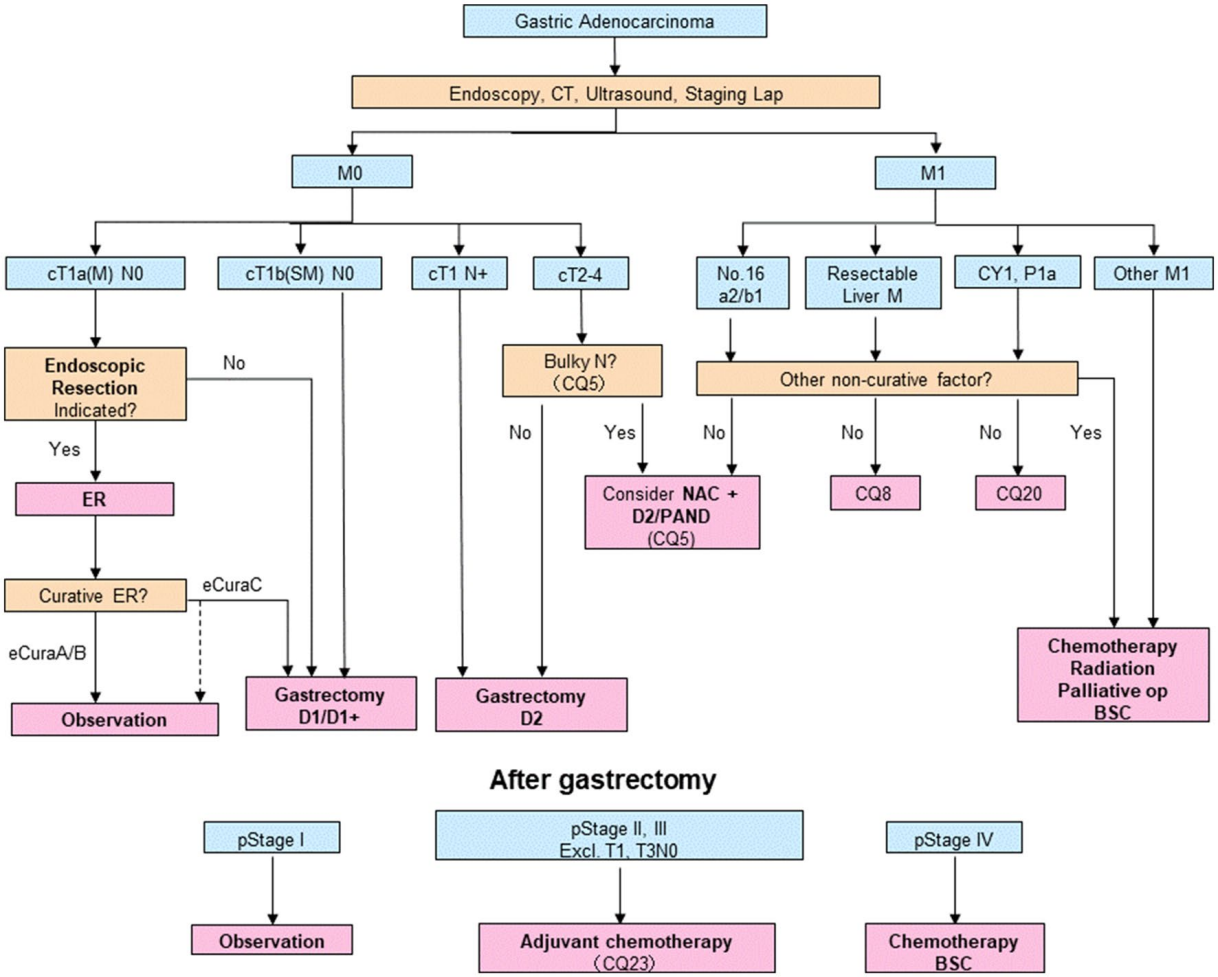
Algorithm of standard treatments. The T/N/M and Stage are used in conjunction with the Japanese Classification of Gastric Carcinoma 15th edition [1] and TNM classification 8th edition [2]

The algorithm is shown in Fig. 1. Description of the tumor status (T/N/M and stage) in this edition is based on the 15th edition of the Japanese Classification of Gastric Carcinoma [1], which is identical to the 8th edition of the International Union Against Cancer (UICC)/TNM Classification [2].

#### Summary of T, N, and M categories and stage grouping based on the 15th edition of the Japanese Classification of Gastric Carcinoma [1]

- N1: the number of metastatic lymph nodes among the regional lymph nodes (No. 1–12. 14v) is 1–2, N2: 3–6, N3a: 7–15, N3b: ≥ 16.
- M1: metastasis outside the regional lymph nodes (including CY1).
- Stage grouping: See Table 1.

**Table 1:** Stage grouping

	M0		M1
	N0	N(+)	any N
Clinical stages (cTNM, cStage, to be decided based on preoperative imaging, staging laparoscopy findings and intraoperative findings)			
T1 (M, SM)/T2 (MP)	I	IIA	IVB
T3 (SS)/T4a (SE)	IIB	III	
T4b(SI)	IVA		

	M0					M1
	N0	N1	N2	N3a	N3b	any N
Pathological stages (pTNM, pStage, to be decided based on pathologic findings of the resected specimen)						
T1a (M)/pT1b(SM)	IA	IB	IIA	IIB	IIIB	IV
T2 (MP)	IB	IIA	IIB	IIIA	IIIB	
T3 (SS)	IIA	IIB	IIIA	IIIB	IIIC	
T4a (S)	IIB	IIIA	IIIA	IIIB	IIIC	
T4b (SI)	IIIA	IIIB	IIIB	IIIC	IIIC

## Surgery

### Types and definitions of gastric surgery

#### Standard gastrectomy and non-standard gastrectomy in surgery with curative intent

##### Standard gastrectomy

**Fig. 2:**
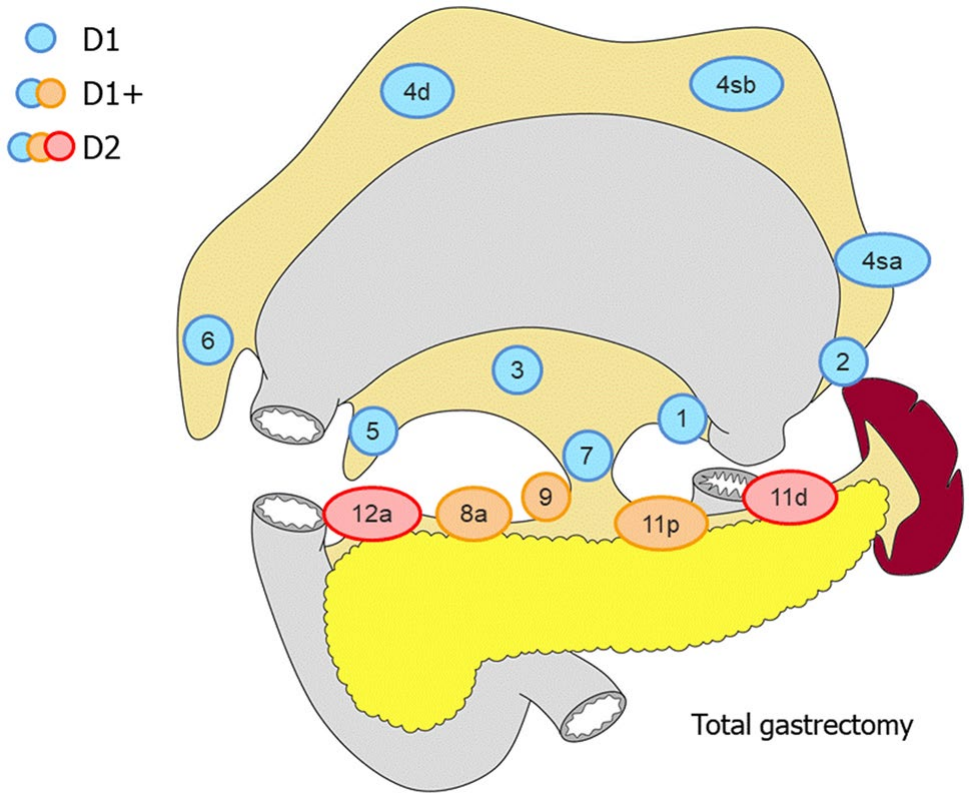
Lymph node dissection in total gastrectomy. Lymph node stations in blue need to be dissected in D1 dissection. In addition, lymph node stations in orange need to be dissected in D1+ dissection and lymph node stations in red as well in D2 dissection

Standard gastrectomy is the principal surgical procedure performed with curative intent. It involves resection of at least two-thirds of the stomach with a D2 lymph node dissection (refer to the section of “lymph node dissection" and Fig. 2 and 5 for the definition of D-categories).

##### Non-standard gastrectomy

In non-standard gastrectomy, the extent of gastric resection and/or lymphadenectomy is altered according to tumor stages. It includes modified surgery and extended surgery.

##### Modified surgery

The extent of gastric resection and/or lymphadenectomy is reduced (D1, D1+, etc.) compared to standard surgery.

##### Extended surgery

(1) Gastrectomy with combined resection of adjacent involved organs. (2) Gastrectomy with extended lymphadenectomy exceeding D2.

#### Non-curative surgery

Non-curative surgery is offered to the patients who are considered to be incurable. It can be semi-classified into either palliative surgery or reduction surgery depending on the aim of surgery.

##### Palliative surgery

Serious symptoms such as bleeding or obstruction may develop in a patient with advanced/ metastatic gastric cancer. Surgery to relieve symptoms may then be considered an option, and palliative gastrectomy or gastrojejunostomy is selected depending on the resectability of the primary tumor and/or surgical risks. Stomach-partitioning gastrojejunostomy has been reported to result in superior function compared to simple gastrojejunostomy [3].

##### Reduction surgery

Reduction surgery is defined as gastrectomy performed for patients harboring incurable factors such as unresectable liver metastasis and peritoneal metastasis, while suffering from no tumor-associated symptoms such as bleeding and obstruction. It aims to prolong survival or to delay the onset of symptoms by reducing tumor volume. However, an international cooperative randomized controlled trial (REGATTA, JCOG0705/KGCA01) failed to prove survival benefit of reduction surgery [4]. Therefore, surgeons are strongly advised not to perform this type of surgery any more (CQ1).

### Extent of gastric resection

#### Surgery for gastric cancer

Surgery for gastric cancer is defined as follows in the order of the stomach volume to be resected.

- *Total gastrectomy* Total resection of the stomach including the cardia and pylorus.
- *Distal gastrectomy* Stomach resection including the pylorus. The cardia is preserved. In the standard gastrectomy, two-third of the stomach is resected.
- *Pylorus*-*preserving gastrectomy* (*PPG*) Stomach resection preserving the upper third of the stomach and the pylorus along with a portion of the antrum.
- *Proximal gastrectomy* (*PG*) Stomach resection including the cardia (esophagogastric junction). The pylorus is preserved.
- *Segmental gastrectomy* Circumferential resection of the stomach preserving the cardia and pylorus.
- *Local resection* Non-circumferential resection of the stomach.
- *Non*-*resectional surgery* (bypass surgery, gastrostomy, jejunostomy).

In addition, surgery for cancer of the gastric remnant is defined as follows.

- *Completion gastrectomy* Total resection of the remnant stomach including the cardia or pylorus depending on the type of previous gastrectomy.
- *Subtotal resection of remnant stomach* Distal resection of the remnant stomach preserving the cardia.

#### Determination of the extent of gastric resection

##### Resection margin

A sufficient resection margin should be ensured when determining the resection line in gastrectomy with curative intent. Proximal margin of at least 3 cm is recommended for T2 or deeper tumors with an expansive growth pattern (types 1 and 2) and 5 cm for those with an infiltrative growth pattern (types 3 and 4). When these rules cannot be satisfied, it is advisable to examine the whole thickness of proximal resection margin by frozen section. For tumors invading the esophagus, resection margin > 5 cm is not necessarily required, but frozen section examination of the resection line is preferable to ensure an R0 resection.

For T1 tumors, a gross resection margin of 2 cm should be obtained. When the tumor border is unclear and difficulties in deciding on the resection line are expected, preoperative endoscopic marking by clips of the tumor border based on the biopsy results would be helpful.

##### Selection of gastrectomy

The standard surgical procedure for clinically node-positive (cN+) or T2–T4a tumors is either total or distal gastrectomy. Distal gastrectomy is selected when a satisfactory proximal resection margin (see above) can be obtained. When obtaining proximal resection margin is not possible, total gastrectomy is selected. Even in a case that a satisfactory proximal resection margin can be obtained, pancreatic invasion by tumor requiring pancreaticosplenectomy necessitates total gastrectomy regardless of the tumor location. Total gastrectomy with splenectomy should be considered for tumors that are located along the greater curvature and harbor metastasis to no. 4sb lymph nodes, even if the primary tumor could be removed by distal gastrectomy. For adenocarcinoma of which major part locates on the proximal side of the esophagogastric junction, esophagectomy of the middle and lower parts of esophagus and proximal gastrectomy with gastric tube reconstruction should be considered, similar to surgery for esophageal cancer.

For cT1N0 tumors, the following types of gastric resection can be considered according to tumor location.

- Pylorus-preserving gastrectomy (PPG): for tumors in the middle portion of the stomach with the distal tumor border at least 4 cm proximal to the pylorus.
- Proximal gastrectomy: for proximal tumors where more than half of the distal stomach can be preserved.
- Local resection of the stomach and segmental gastrectomy should still be regarded as investigational treatments.

### Lymph node dissection

#### Extent of lymph node dissection

The extent of lymphadenectomy is classified by the D-level criteria into D1, D1+ or D2, and is defined as follows according to the type of gastrectomy conducted. The indications for each of the D levels are described in the subsequent section. See descriptions under the title “Junctional cancer” for the current recommendations on the extent of lymph node dissection for the esophagogastric junctional carcinoma.

#### Definition of the D levels

The extent of systematic lymphadenectomy is defined as follows, according to the type of gastrectomy conducted. When the extent of lymphadenectomy performed does not fully comply with the D-level criteria, the lymph node station that has been additionally resected or left in situ could be recorded as in the following examples: D1 (+ No. 8a), D2 (− No. 12a). However, when registering data to the nationwide database, the D levels need to be strictly determined and should be downgraded in case resection of any of the lymph node stations that should have been resected to fulfill the criteria of a D level was omitted. (e.g., D2(− No. 12a) should be registered as D1+).

##### Total gastrectomy (Fig. 2)

- D0: Lymphadenectomy less than D1.
- D1: No. 1–7.
- D1+: D1 + No. 8a, 9, 11p.
- D2: D1 + No. 8a, 9, 11p, 11d, 12a.

For tumors invading the esophagus, resection of No. 110* should be added to D1+, and resection of Nos. 19, 20, 110* and 111 to D2.

##### Distal gastrectomy (Fig. 3)

- D0: Lymphadenectomy less than D1.
- D1: No. 1, 3, 4sb, 4d, 5, 6, 7.
- D1+: D1 + No. 8a, 9.
- D2: D1 + No. 8a, 9, 11p, 12a.

**Fig. 3:**
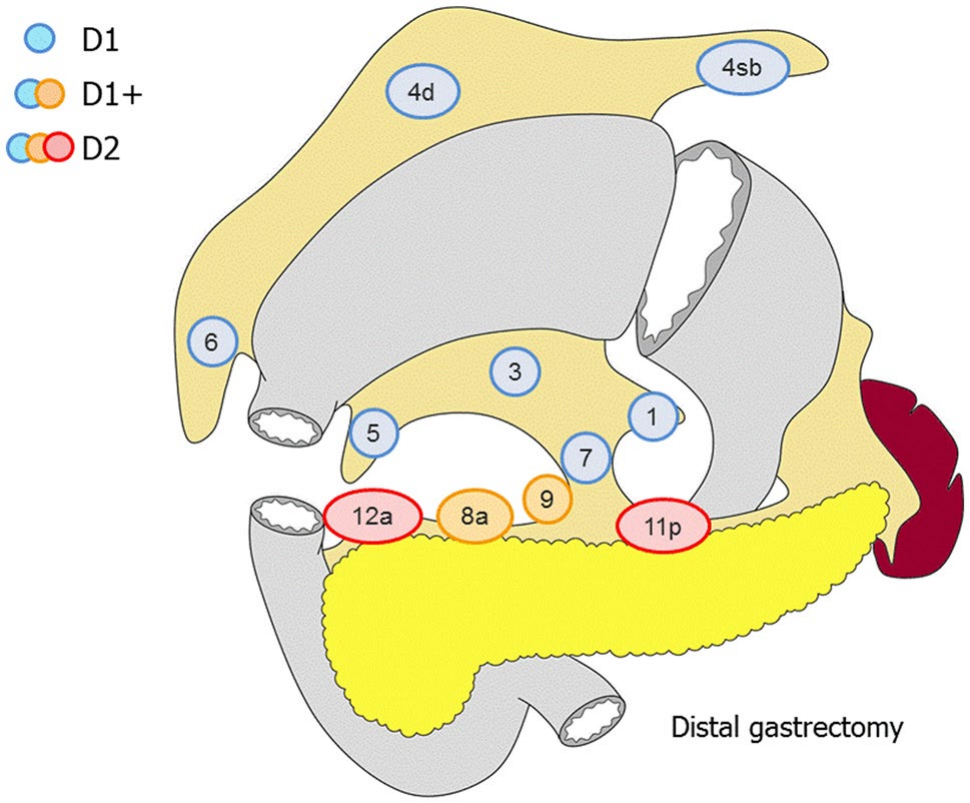
Lymph node dissection in distal gastrectomy. Lymph node stations in blue need to be dissected in D1 dissection. In addition, lymph node stations in orange need to be dissected in D1+ dissection and lymph node stations in red as well in D2 dissection

##### Pylorus-preserving gastrectomy (Fig. 4)

- D0: Lymphadenectomy less than D1.
- D1: No. 1, 3, 4sb, 4d, 6, 7.
- D1+: D1 + No. 8a, 9.

**Fig. 4:**
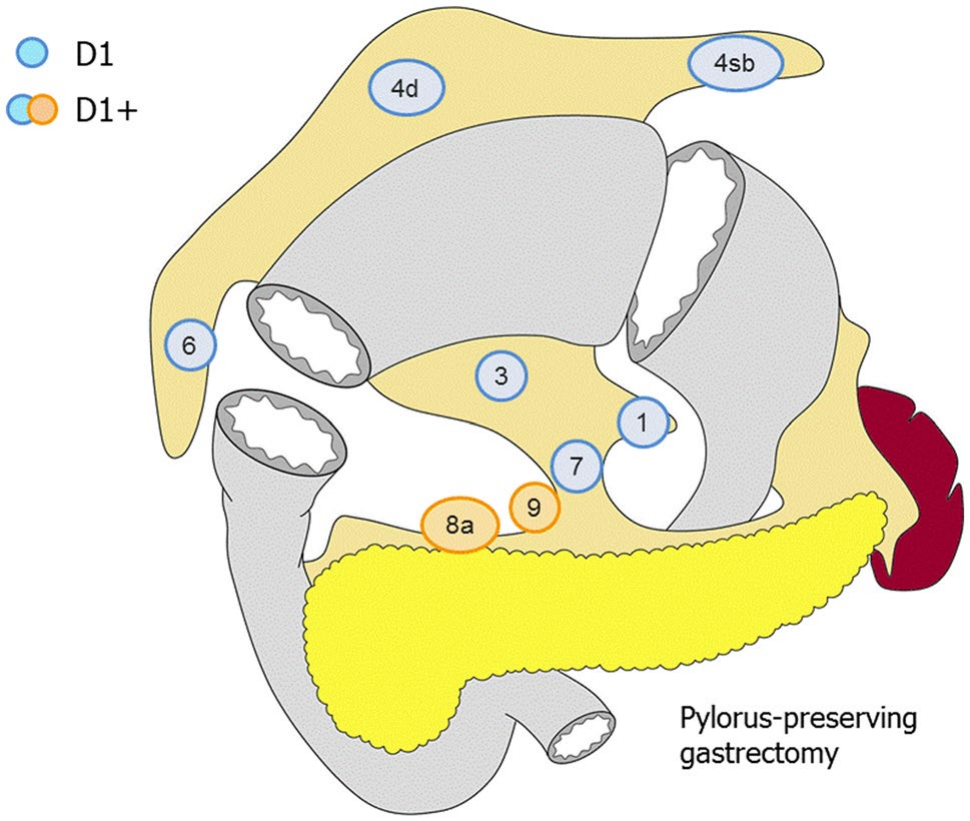
Lymph node dissection in pylorus-preserving gastrectomy. Lymph node stations in blue need to be dissected in D1 dissection. In addition, lymph node stations in orange need to be dissected in D1+ dissection

##### Proximal gastrectomy (Fig. 5)

- D0: Lymphadenectomy less than D1.
- D1: No. 1, 2, 3a, 4sa, 4sb, 7
- D1+: D1 + No. 8a, 9, 11p.

**Fig. 5:**
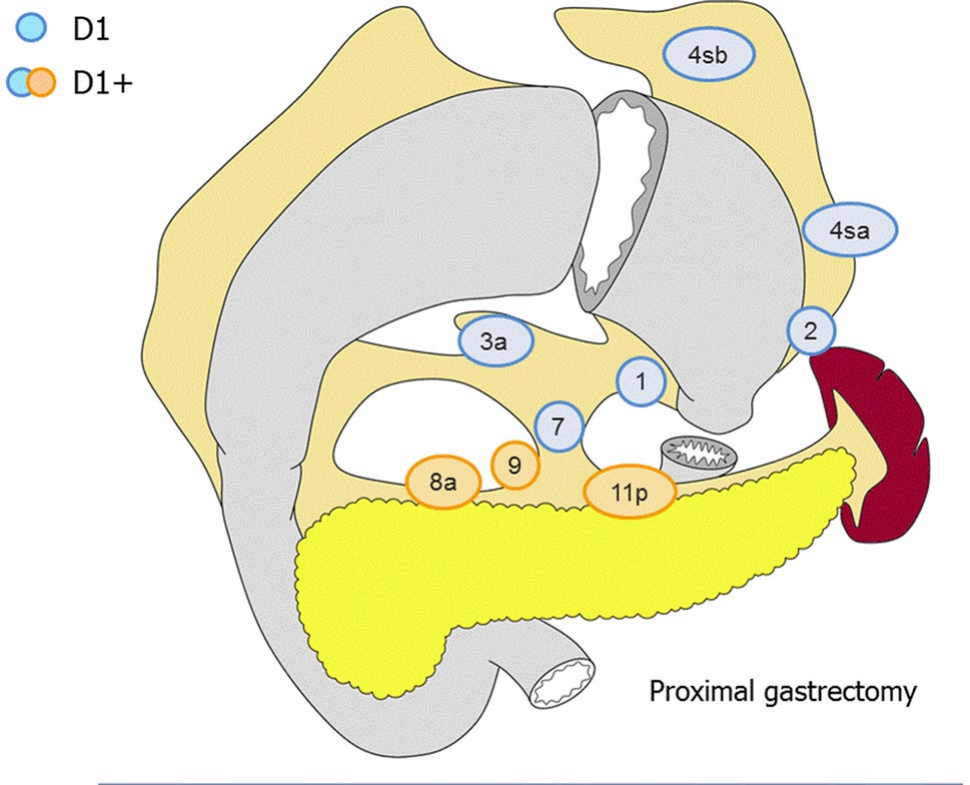
Lymph node dissection in proximal gastrectomy. Lymph node stations in blue need to be dissected in D1 dissection. In addition, lymph node stations in orange need to be dissected in D1+ dissection

For tumors invading the esophagus, No. 110*should additionally be dissected in D1+.

*No. 110 lymph nodes (lower thoracic para-esophageal nodes) in gastric cancer invading the esophagus are those attached to the lower part of the esophagus that is removed to obtain a sufficient resection margin. When esophagectomy and proximal gastrectomy are performed for esophagogastric junctional carcinoma (Ae), the definition of No. 110 lymph nodes complies with the definition in the Japanese Classification of Esophageal Cancer.

#### Indications for lymph node dissection

In principle, D2 lymphadenectomy is indicated for cN+ or ≥ cT2 tumors and a D1 or D1+ for cT1N0 tumors. Since pre- and intraoperative diagnoses regarding the depth of tumor invasion and nodal involvement remain unreliable, D2 lymphadenectomy should be performed whenever the possibility of nodal involvement cannot be dismissed.

##### D1 lymphadenectomy

A D1 lymphadenectomy is indicated for cT1a tumors that do not meet the criteria for EMR/ESD, and for cT1bN0 tumors that are histologically of differentiated type and 1.5 cm or smaller in diameter.

##### D1+ lymphadenectomy

A D1+ lymphadenectomy is indicated for cT1N0 tumors other than the above.

##### D2 lymphadenectomy

A D2 lymphadenectomy is indicated for potentially curable cT2–T4 tumors as well as cT1N+ tumors. Spleen should be preserved in total gastrectomy for advanced cancer of the upper stomach provided the tumor does not involve the greater curvature [5] (CQ4). The role of splenectomy for tumors invading the greater curvature remains equivocal.

##### D2+ lymphadenectomy

Gastrectomy with extended lymphadenectomy beyond D2 is classified as a non-standard gastrectomy, and could be considered for the following cases although hard evidence is lacking, on the condition that it can be conducted safely.

- Dissection of No. 10 (splenic hilar lymph nodes) with or without splenectomy for cancer of the upper stomach invading the greater curvature (D2 + No. 10). This procedure had been defined as D2 lymphadenectomy in the previous editions of the Japanese Gastric Cancer Treatment Guidelines (CQ4).
- Dissection of No. 14v (superior mesenteric venous lymph node) for cancer of the distal stomach tumor with metastasis to the No. 6 lymph nodes (D2 + No. 14v).
- Dissection of No. 13 (posterior pancreas head lymph node) for cancer invading the duodenum (D2 + No. 13) [6]. Metastases to the No. 13 nodes, which are not included in the regional lymph nodes for gastric cancer, should usually be classified as M1. However, since the No. 13 nodes are among the regional lymph nodes for cancer of the duodenum according to the TNM classification and the Japanese Classification of Gastric Carcinoma 15th edition, these should be regarded as regional lymph nodes once gastric cancer invades the duodenum.
- Dissection of No. 16 (abdominal aortic lymph node) after neoadjuvant chemotherapy for cancer with an extensive lymph node involvement (D2 + No. 16) (CQ5).

#### Junctional cancer (diameter less than 4 cm)

**Fig. 6:**
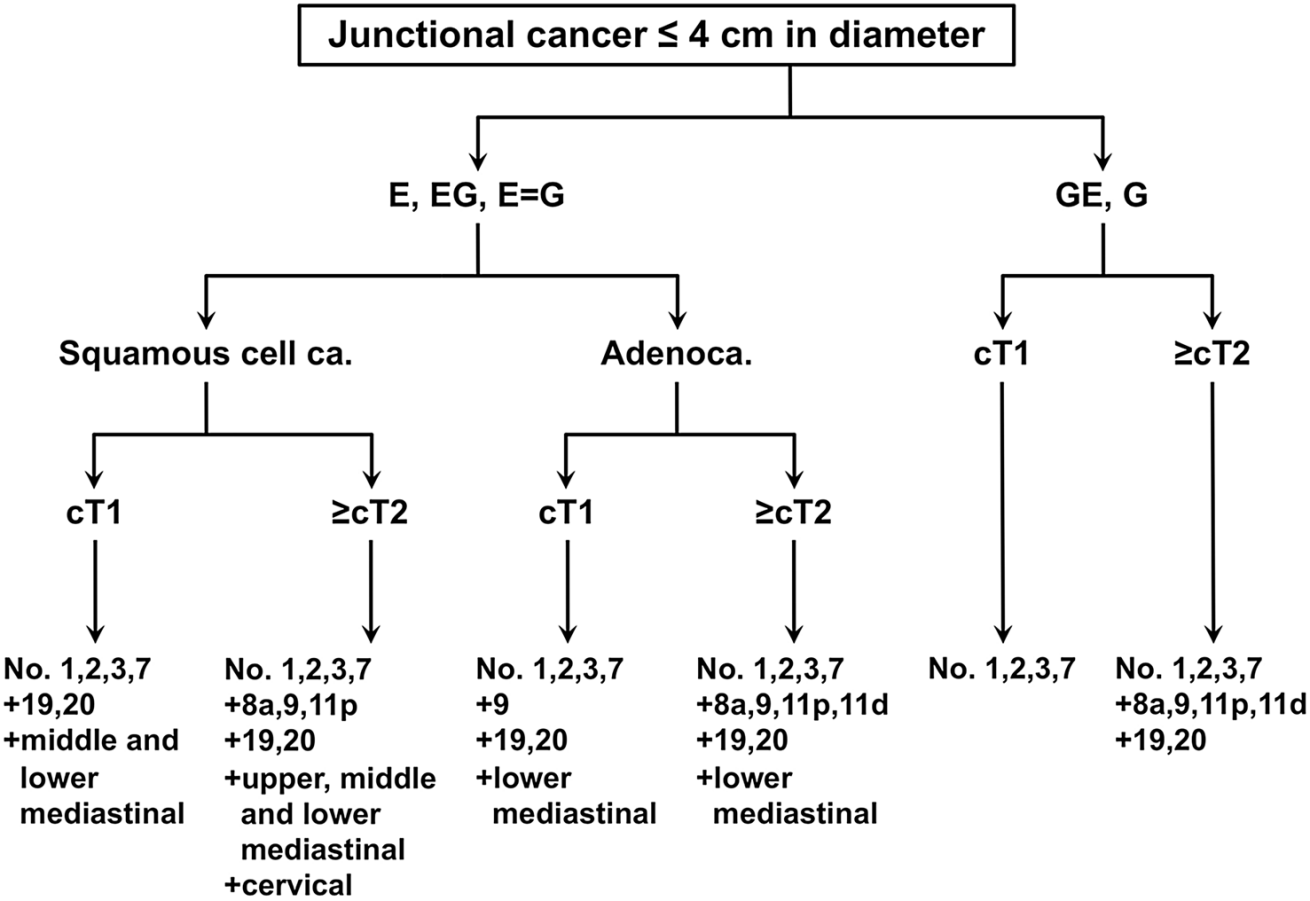
Algorithm of lymph node dissection for junctional carcinoma with diameter ≤ 4 cm. Difficulties are expected in accurately discriminating between lymph node station Nos. 19 and 20 and among lymph node station Nos. 110, 111 and 112. Thus, the lymph node around the hiatus and lower mediastinal lymph nodes are expected to be removed en bloc. Complete removal of lymph node station No. 3b is not mandatory when proximal gastrectomy is selected. 1) Clinical relevance of dissecting the upper mediastinal lymph nodes is unclear since the incidence of metastasis is low. 2) Cervical lymph nodes are infrequently dissected and clinical relevance of dissecting these nodes is unknown. However, it is noteworthy that there are long-term survivors among those with histologically confirmed metastases among the cervical nodes. 3) For the E=G category, lower mediastinal modes and hiatal nodes were rarely dissected, and the incidence of metastasis among those who underwent resection was low. 4) Cervical, upper mediastinal and middle mediastinal nodes are rarely dissected for this category, and data to discuss on the clinical relevance of dissecting these nodes are lacking

The current edition of the Japanese Gastric Cancer Treatment Guidelines defines the extent of lymphadenectomy according to the type of gastrectomy regardless the tumor location. However, only for esophagogastric junctional cancer (adenocarcinoma or squamous cell carcinoma), of which center locates within 2 cm of the esophagogastric junction, there is no consensus over the type of resection and the extent of lymphadenectomy that could be a standard of care for this category. In 2012–2013, the Japanese Gastric Cancer Association and Japan Esophageal Society joined forces to conduct a nationwide surveillance of junctional cancer of less than 4-cm diameter, and retrospective data of 3177 patients operated on between 2001 and 2010 were collected from 273 institutions [7]. An algorithm showing the tentative standard in the extent of lymphadenectomy based on the tumor location, histology and T-categories was constructed based on this surveillance (Fig. 6). A prospective phase II study by the same joint force to further investigate this issue is on-going.

#### Extent of the resection of the esophagus and stomach

Either of the following procedures is selected: proximal gastrectomy with or without lower esophageal resection, total gastrectomy with or without lower esophageal resection, esophageal resection and upper gastric resection.

#### Extent of lymphadenectomy

Lymphadenectomy as shown in the algorithm is the tentative recommendation, although survival benefit of more extensive lymphadenectomy cannot be denied at this time. D levels and resected lymph nodes should be recorded according to the current classification for total gastrectomy or proximal gastrectomy as shown in Fig. 2 and 5. Refer also to the section “Lymph node dissection”. For example, in case of total gastrectomy, when lower esophageal resection and lymphadenectomy as indicated in the algorism (dissection of Nos. 1, 2, 3, 7, 8a, 9, 11p, 11d, 19, 20) are performed for cT3 esophagogastric junctional carcinoma of 3.0-cm diameter, the D level should be recorded as D2 (− No. 110, 111).

### Miscellaneous

#### Vagal nerve preservation

It is reported that preservation of the hepatic branch of the anterior vagus and/or the celiac branch of the posterior vagus contributes to improving postoperative quality of life through reducing post-gastrectomy gallstone formation, diarrhea and/or weight loss. In case of PPG, the hepatic branch should be preserved to maintain the pyloric function (CQ2).

#### Omentectomy

Removal of the greater omentum is usually integrated in the standard gastrectomy for T3 or deeper tumors. For T1/T2 tumors, the omentum more than 3 cm away from the gastroepiploic artery may be preserved.

#### Bursectomy

Bursectomy for tumors penetrating the serosa of the posterior gastric wall had been performed with the aim of removing microscopic tumor deposits within the omental cavity. However, survival benefit of this procedure has been denied by a large-scale randomized trial (JCOG1001), not only for all patients registered but also for subsets with T4a tumors and tumors located in the posterior wall [8].

#### Combined resection of adjacent organ(s)

For tumors in which the primary or metastatic lesion directly invades adjacent organs, combined resection of the involved organ may be performed to obtain an R0 resection.

#### Approaches to the lower esophagus

For gastric cancers invading less than 3 cm of the distal esophagus, a transhiatal abdominal approach is recommended (JCOG9502) [9]. Where a greater length of esophagus is involved, a transthoracic approach should be considered if the surgery is potentially curative.

#### Laparoscopic surgery

Laparoscopic surgery can be considered as an option to treat cStage I cancer that is resectable by distal gastrectomy. In the 2014 version of the guidelines by the Japan Society for Endoscopic Surgery, distal gastrectomy by the laparoscopic approach was recommended for gastric cancer with the diagnosis of cStage I, according to the Japanese Classification of Gastric Carcinoma 14th edition (rated recommendation B). These decisions reflect the fact that superiority of the laparoscopic approach in terms of short-term outcome has been reported through small-scale randomized trials and meta-analyses, while safety was proven in a prospective phase II study (JCOG0703) conducted by certified surgeons with sufficient skill and knowledge in laparoscopic surgery [10]. However, surgeons will have to be aware that the learning curve exists, and the indication for this approach should, therefore, be decided discreetly in each institution based on the expertise of the surgical team.

Data regarding the long-term outcome are yet to be available, and results of pivotal phase III studies conducted in Japan (JCOG0912 [11]) and Korea (KLASS01 [12]) are awaited*. As for more advanced cancer, there is currently no evidence to recommend a laparoscopic approach, since randomized trials to look at safety and long-term outcome are currently ongoing (JLSSG0901) [13].

No prospective trial has been reported regarding total gastrectomy for early gastric cancer by the laparoscopic approach**. Thus, laparoscopic total gastrectomy has been rated by the guidelines of the Japan Society for Endoscopic Surgery (2014) as recommendation C1 (may be considered for a patient in need of total gastrectomy, but no scientific evidence in support of the procedure is currently available) (CQ7). Those who consider to try this procedure in their institution should plan to do so with sufficient caution, since postoperative complications were reported to be significantly more frequent in the first year of its introduction.

When conducting gastrectomy by the laparoscopic approach, informed consent should be obtained from all patients after providing sufficient information, including the lack of data regarding long-term consequences.

*Survival data from the KLASS01 trial are now available [14]. Intention-to-treat analysis confirmed non-inferiority of the laparoscopic approach compared with open approach, the 5-year overall survival being 94.2% in the laparoscopic group and 93.3% in the open surgery group.

**Regarding the safety issue of laparoscopy-assisted total or proximal gastrectomy, evidence from a single-arm (JCOG1401) is now available. In this trial, incidence of the Grade 2–4 esophagojejunal anastomotic leakage was 2.5% (6/244, 95% CI 0.9–5.3) and met the required level of safety [15].

### Reconstruction after gastrectomy

The following reconstruction methods are usually employed. Each has advantages and disadvantages. Functional benefits of the pouch reconstruction are yet to be established.

#### Total gastrectomy

- Roux-en-Y esophagojejunostomy.
- Jejunal interposition.
- Double tract method.

#### Distal gastrectomy

- Billroth I gastroduodenostomy.
- Billroth II gastrojejunostomy.
- Roux-en-Y gastrojejunostomy.
- Jejunal interposition.

#### Pylorus-preserving gastrectomy

- Gastro-gastrostomy.

#### Proximal gastrectomy

- Esophagogastrostomy.
- Jejunal interposition.
- Double tract method.

## Endoscopic resection

### Methods of endoscopic resection (CQ11)

#### Endoscopic mucosal resection (EMR)

The lesion, together with the surrounding mucosa, is lifted by submucosal injection of saline (normo- or hypertonic) and removed using a high-frequency steel snare [16, 17].

#### Endoscopic submucosal dissection (ESD)

The mucosa surrounding the lesion is circumferentially incised using a high-frequency electric knife (usually insulation-tipped), and the submucosal layer is dissected from the proper muscle layer [18–20].

### Handling of endoscopically resected specimens

#### Handling of resected specimens

The resected specimens should be handled according to the rules described in the Japanese Classification of Gastric Carcinoma 15th edition [1].

#### Definition of differentiated-type and undifferentiated-type carcinoma

The tumor biopsy specimens and endoscopically resected tumors are histologically classified into either the differentiated or undifferentiated type. The former includes malignant epithelial tumor, general type, of papillary adenocarcinoma (pap) and tubular adenocarcinoma (tub1, tub2), and the latter includes that of poorly differentiated adenocarcinoma (por1, por2) and signet ring cell carcinoma (sig) according to the Japanese Classification of Gastric Carcinoma 15th edition. In case mucinous adenocarcinoma (muc) was found at the submucosal layer, resected specimen is handled as undifferentiated type, regardless of whether it is considered to derive from the differentiated or undifferentiated type.

#### Histological predominance and intratumoral ulcerative findings (UL)

A tumor consisting of components of both differentiated- and undifferentiated-type carcinoma is, nevertheless, classified into one of the two types according to the quantitative predominance. In addition, when more than one histological type is found in a tumor, all histological types are to be recorded in the order of quantitative predominance, e.g., tub2 > tub1. Diagnosis of UL1 is principally made based on the histological evidence of ulcerative findings. However, the histological diagnosis of UL is sometimes difficult because of a biopsy-derived scar. Thus, endoscopic and/or radiological evidence should also be taken into consideration when making a conclusive diagnosis. A biopsy-derived scar is usually observed histologically as fibrosis restricted to small areas just beneath the muscularis mucosae [21]. If it cannot be discriminated from the ulcer scar, it should be classified as UL1.

### Indication for endoscopic resection

Lesions that could technically be resected by endoscopy are classified into the following three categories depending on the strength of evidence. “A tumor indicated for endoscopic resection as a standard treatment (absolute indication)” is defined as a tumor in which a possibility of harboring lymph node metastasis is less than 1%. For this population, endoscopic resection is expected to have therapeutic effect equivalent to a surgical resection. “A tumor indicated for endoscopic resection as an investigational treatment (expanded indication)” is defined as a tumor in which sufficient evidence for long-term outcome after endoscopic resection is lacking, although a possibility of harboring lymph node metastasis is less than 1%. “A tumor indicated for endoscopic resection as clinical practice under some circumstances (relative indication)” is defined as a tumor which would usually be treated by surgical resection, but for which endoscopic resection may still lead to cure and could, therefore, be an option when surgery cannot be recommended due to various clinical circumstances.

#### Principles of indication

Endoscopic resection is considered for tumors that have a very low possibility of lymph node metastasis and are suitable for en bloc resection [22].

#### Indication

*Absolute indication*

*Absolute indication of EMR or ESD* [23, 24]

A differentiated-type adenocarcinoma without ulcerative findings (UL0), in which the depth of invasion is clinically diagnosed as T1a and the diameter is ≤ 2 cm.

*Absolute indication of ESD*

- A differentiated-type adenocarcinoma without ulcerative findings (UL0), in which the depth of invasion is clinically diagnosed as T1a and the diameter is > 2 cm.
- A differentiated-type adenocarcinoma with ulcerative findings (UL1), in which the depth of invasion is clinically diagnosed as T1a and the diameter is ≤ 3 cm.

*Expanded indication* [25]

- An undifferentiated-type adenocarcinoma without ulcerative findings (UL0) in which the depth of invasion is clinically diagnosed as T1a and the diameter is ≤ 2 cm. Lesions in this category are currently excluded from the absolute indication due to the lack of sufficient evidence for long-term outcome, but may in future be included pending results of the JCOG1009/1010 study.

*Relative indication*

A standard therapy is surgical resection for tumors that do not fulfill the absolute or expanded indications. However, endoscopic resection could be an option for the elderly and high-operative-risk patients with severe comorbidities. Such case is considered as a relative indication, and endoscopic resection could be performed, provided a consent was obtained from the patient after explaining the risk of residual disease, possibly in the form of lymph node metastasis.

*Indication of endoscopic resection for local recurrence after EMR/ESD* [26]

Local recurrence within the mucosa after initial EMR/ESD for tumors that had fulfilled the absolute indication could be considered as expanded indication for repeat endoscopic resection. However, given paucity of hard evidence in support of the repeat endoscopic resection, a large-scale observational study looking at the long-term outcome of this procedure is warranted.

### Curability of endoscopic resection

#### Evaluation of curability

Two factors should be considered for curability assessment: completeness of the primary tumor removal and possibility of lymph node metastasis.

#### Endoscopic curability A (eCuraA)

The resection is classified as endoscopic curability A (eCuraA) when all of the following conditions are fulfilled, provided cancer is without ulcerative findings (UL0): en bloc resection, any tumor size, histologically differentiated type-dominant, pT1a, negative horizontal margin (HM0), negative vertical margin (VM0) and no lymphovascular infiltration (Ly0, V0). However, if the undifferentiated component of the lesion exceeds 2 cm in length, the endoscopic curability is classified as C-2 (eCuraC-2).

When cancer is with ulcerative findings (UL1), the resection is still classified as eCuraA when all of the following conditions are fulfilled: en bloc resection, tumor size ≤ 3 cm, histologically differentiated type-dominant, pT1a, HM0, VM0, Ly0, V0.

#### Endoscopic curability B (eCuraB)

The resection is classified as endoscopic curability B (eCuraB) for histologically undifferentiated type-dominant when all of the following conditions are fulfilled: UL0, en bloc resection, pT1a, HM0, VM0, Ly0, V0, tumor size ≤ 2 cm.

The resection is also classified as endoscopic curability B (eCuraB) for pT1b cancer when all of the following conditions are fulfilled: en bloc resection, histologically of differentiated type-dominant, pT1b1 (SM1) (< 500 μm from the muscularis mucosae), HM0, VM0, Ly0, V0, tumor size ≤ 3 cm. However, if the undifferentiated component is included in the portion of submucosal invasion, the endoscopic curability is classified as C-2 (eCuraC-2) [27].

#### Endoscopic curability C (eCuraC)

The resection is classified as endoscopic curability C (eCuraC) when it does not fulfill the conditions described above to be classified as either eCuraA or eCuraB.

The resection is classified as endoscopic curability C-1 (eCuraC-1) when it is histologically differentiated type and fulfills other criteria to be classified into either eCuraA or eCuraB but was either not resected en bloc or had positive horizontal margin. All other eCuraC resections are subclassified as endoscopic curability C-2 (eCuraC-2).

### Treatments after endoscopic resection (Fig. 7)

Treatments should be planned as follows after evaluation of curability based on the histological examination of the resected specimens.

**Fig. 7:**
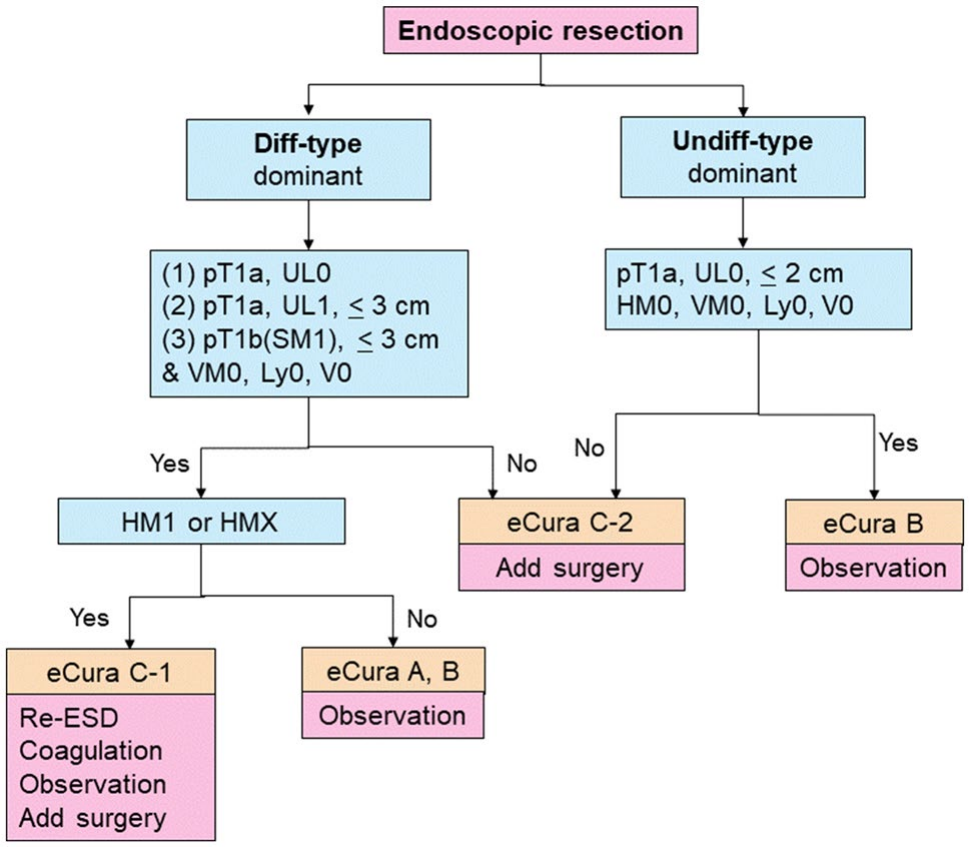
Algorithm showing curability decision and additional treatments for patients who underwent endoscopic resection

#### Treatments after eCuraA or eCuraB

Follow-up with annual or biannual endoscopy is recommended after the eCuraA resection [28]. In addition, follow-up with abdominal ultrasonography or computed tomography (CT) for surveillance of metastases is recommended after the eCuraB resection [29, 30]. For both eCuraA and eCuraB resection, it has been recommended that *Helicobacter pylori* be examined and, if positive, be eradicated (CQ12). However, some studies showed that the Helicobacter eradication after endoscopic resection had no impact on the occurrence of metachronous cancer. Further investigations regarding this issue are warranted [31, 32].

#### Treatments after eCuraC-1

Since the risk for harboring lymph node metastasis is low, one of the following alternatives could be selected according to the institutional policy after obtaining patient’s consent: repeat ESD, surgical resection, close observation expecting a burn effect of the initial ESD, and endoscopic coagulation using a laser or argon-plasma coagulator [33].

When the lesion is differentiated type of ≤ 3 cm and either UL1or pT1b1 (SM1), size of the residual mucosal lesion should be reassessed by endoscopy. When the sum of the lengths of the resected and residual lesions exceeds 3 cm, gastrectomy with lymphadenectomy should be considered the standard of care. In addition, patients with positive horizontal margin within the portion of submucosal invasion and those who underwent piecemeal resection in which the resection line involved the portion of submucosal invasion should be recommended to undergo gastrectomy with lymphadenectomy, since the histological diagnosis under these circumstances is destined to be uncertain.

#### Treatments after eCuraC-2

Gastrectomy with lymphadenectomy should be considered as the standard of care. When surgery cannot be recommended because of old age or severe comorbidities, the risk of residual disease in the form of lymph node metastasis (Tables 2 [34] and 3 [35]) and possibility of the subsequent local recurrence and/or distant metastasis should be assessed and explained sufficiently to the patients, along with the information that the recurrent disease is usually incurable with dismal prognosis.

**Table 2:**
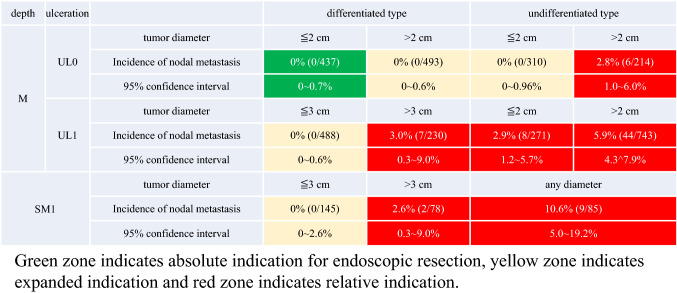
Incidence of nodal metastasis in various categories of early gastric cancer observed from surgically resected specimens operated at National Cancer Center Hospital and Cancer Institute Hospital [34]

Green zone indicates absolute indication for endoscopic resection, yellow zone indicates expanded indication and red zone indicates relative indication

**Table 3:** The incidence of nodal metastasis observed from the specimens of patients who underwent additional gastrectomy with lymphadenectomy after initial treatment with endoscopic resection

Total points	Number of patients (*n *= 1101)	Number of patients with lymph node metastasis (*n *= 94)	Incidence of nodal metastasis (%)
0	62	1	1.6
1	341	9	2.6
2	185	9	4.9
3	148	11	7.4
4	132	11	8.3
5	141	28	19.9
6	77	21	27.3
7	15	4	26.7

Total points refer to the total of following scoring scheme: one point added to each of the following findings: diameter ≥ 3 cm, positive vertical margin, venous invasion, depth ≥ SM2. Three points added to a histopathologic finding of lymphatic invasion [35]

## Systemic chemotherapy for unresectable advanced/recurrent gastric cancer (AGC) (CQ13–CQ22)

Although recent advances in chemotherapy have achieved considerable tumor shrinkage in many cases of AGC, these responses have not ultimately led to cure. The median survival time achieved in domestic and international clinical trials for the disease at this stage remains 6–14 months [36, 37]. The current goal of chemotherapy, therefore, is to delay the manifestation of, or ameliorate, the disease-related symptoms and to prolong survival.

Clinical benefits of chemotherapy have been proven in randomized controlled trials comparing chemotherapy with best supportive care (BSC) in patients with performance status (PS) of 0–2, with overall survival as the primary endpoint [38–40]. Although very rare, some patients with AGC actually survive more than 5 years. Thus, systemic chemotherapy is the treatment to be primarily considered for patients with AGC or those who underwent non-curative (R2) resection.

## Principles of indication of systemic chemotherapy for AGC

Systemic chemotherapy is indicated for patients with AGC or those who underwent R2 resection, provided general condition and major organ functions are preserved. To be more specific, patients of PS 0–2 with either unresectable locally advanced cancer or cancer with synchronous or metachronous distant metastases are indicated.

### Standard criteria for a patient to be indicated for systemic chemotherapy

Upon administration of chemotherapy, the indication should be decided for each patient by checking into the following items.

- Histologically proven gastric cancer
- PS 0–2. Chemotherapy is generally not recommended for patients with PS 3 or worse, and the decision to make an exception to the rule should be made discreetly considering the safety and clinical consequences for each individual (safety is of a particular concern for AGC with massive ascites or extensive peritoneal metastases).
- Preserved major organ function
- No serious comorbidities
- Written informed consent obtained from the patient

#### Routine evaluations before and during chemotherapy

1. The following items should be checked or measured prior to initiation of chemotherapy: PS, height, weight, symptoms, medical examination findings, laboratory data including hepatitis virus tests, and the size of tumor lesions assessed by computed tomography (CT) or other appropriate diagnostic modalities.
2. Response should be assessed by appropriate modalities including CT, gastrointestinal endoscopy, and contrast X-ray examination every 2 or 3 months, comparing the diagnostic findings with the corresponding data obtained prior to initiation of chemotherapy or at the best response. Tumor response should be evaluated by the Japanese Classification of Gastric Carcinoma or the Response Evaluation Criteria in Solid Tumors (RECIST) to decide whether or not to continue with the ongoing chemotherapy.
3. The decision of whether or not to continue with the treatment, to modify the drug dosage, or to change the treatment intervals should be made by discreetly balancing the adverse events with efficacy, and referring to the details of clinical trials through which the treatment was approved or otherwise considered beneficial. Care should be taken not to neglect cumulative toxicities such as skin toxicities, dysgeusia and peripheral neuropathy.
4. Appropriate measures should be taken according to the guidelines for reactivation of human hepatitis B virus to deliver chemotherapy for human hepatitis B virus carriers and infected patients (ref: http://www.jsh.or.jp/files/uploads/HBV_GL_ver3_Sep13.pdf, in Japanese).

#### Anti-cancer agents

The following chemotherapeutic or molecular targeted agents are administered in chemotherapy for AGC: fluorouracil (5-FU), tegafur/ 5-chloro-2,4-dihydroxypyridine/potassium oxonate (S-1), levofolinate calcium, capecitabine, cisplatin, oxaliplatin, irinotecan, docetaxel, paclitaxel, nab-paclitaxel, trastuzumab, ramucirumab, and nivolumab. These agents are used either as monotherapy or combination therapy based on the evidence obtained through clinical trials.

### Definition of the recommendation grade and evidence level endowed to each chemotherapeutic regimen

The recommendation grade endowed to each chemotherapeutic regimen is classified into the following two levels, taking into consideration not only evidence from clinical studies but also observations from clinical practice in Japan.

#### “Recommended regimens”

Recommended regimens are defined in this guideline as those that fulfill either of the following requirements for patients who are in sufficient general condition to meet inclusion criteria of clinical trials.

- Significant superiority over, or non-inferiority to, the conventional standard treatment in terms of overall survival has been proven by a domestic or international phase III clinical trial.
- Reproducible clinical benefit has been demonstrated by multiple domestic or international phase II clinical trials for a specific patient group.
- The regimen has served as a control arm in multiple domestic or international phase III clinical trials, and has been considered as one of the standard regimens.

#### “Conditionally recommended regimens”

Conditionally recommended regimens are defined as those that fulfill either of the following requirements and could substitute for the “Recommended regimens” when deemed more appropriate after considering the factors such as i) general condition of the patient including disease status, age, organ functions and comorbidities, ii) social factors such as the necessity for hospital admission, cost of the treatment, distance to the hospital that limits the frequency of visit, and iii) personal preference that derives from the type of adverse events.

- The regimen is considered as having clinical benefit under a specific condition in which the patient may not tolerate the “Recommended regimen”.
- The regimen is considered as having shown clinical benefit based on the wide usage in Japan as general practice or through interpretation of relevant clinical trials, even though the evidence is not robust enough for inclusion into the “Recommended regimen”.

**Fig. 8:**
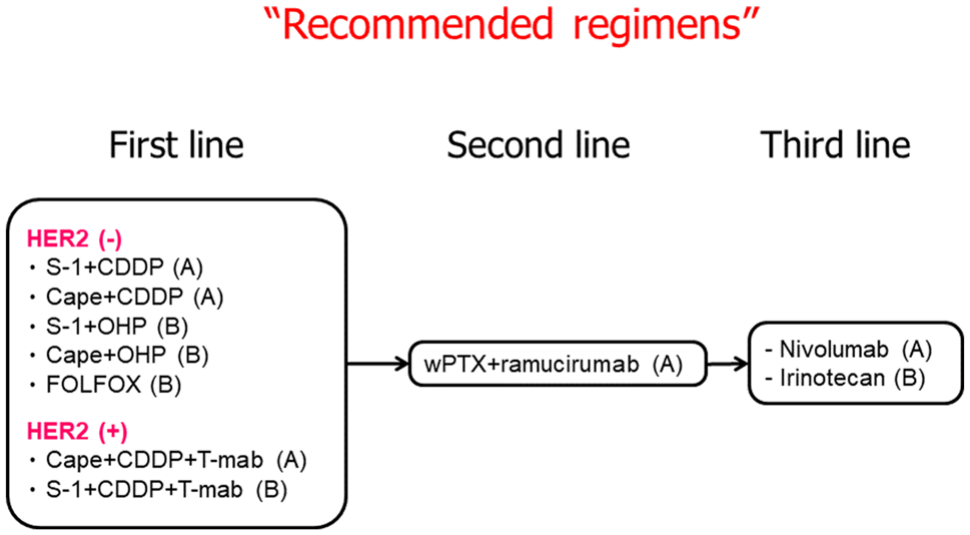
Recommended regimens for the first-, second- and third-line treatments. Only the “Recommended regimens” as defined in the text are included. These regimens are recommended for patients who are in sufficiently favorable general condition to be eligible in the clinical trials from which the evidence in support of these regimens were generated. Strengths of the evidence level for each regimen are shown in brackets

**Fig. 9:**
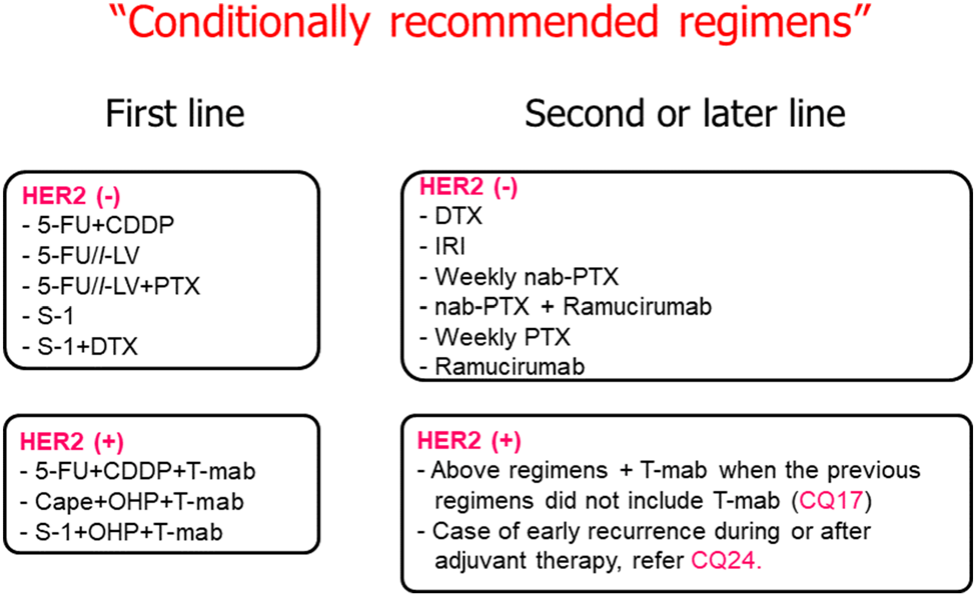
“Conditionally recommended regimens” shown in alphabetical order. Even when using the “Conditionally recommended regimens”, refer to Fig. 8 for the basic strategy and attempt to use drugs from all of the following six categories during the course of the treatment; fluoropyrimidines, platinum, taxanes, irinotecan, ramucirumab and nivolumab. However, it is important to note that continuation of any of the drugs cannot be recommended beyond progression

The “Recommended regimens” and “Conditionally recommended regimens” are listed in Figs. 8 and 9 based on the voting from six medical oncologists who were members of the JGCA Guidelines Committee (the decision supported by at least 70% [5 out of 6] of the medical oncologists). However, the readers are not necessarily discouraged from using regimens which are not listed in these figures. The selection was stringent, and even the regimens which were supported by 50–69% [4 out of 6] of the medical oncologists are not listed. Given the complexity of daily clinical practice, there could be situations where regimens that are not listed could, nevertheless, serve as a useful option.

Due to paucity of clinical trial results specific for elderly patients and for patients with impaired organ function or comorbidities, it is not possible to indicate with sufficient evidence whether a “conditionally recommended regimen” is superior to or safer than a “recommended regimen” delivered at a reduced dosage or modified treatment interval. Therefore, the optimal therapeutic regimen for these patients should be selected on a case-by-case basis, with the guidelines serving only as a reference.

The evidence level according to the criteria of the MINDS clinical guideline manual version 2.0 (Table 4) was provided only for the “Recommended regimens”. For the “Conditionally recommended regimens”, the evidence level is not described because no evidence is available for the specific clinical condition where the “Conditionally recommended regimens” could be more appropriate than the “recommended regimens”.

**Table 4:** Definition of the evidence level

A (strong)	Strong reliability in the expected value of the effect
B (moderate)	Moderate reliability in the expected value of the effect
C (modest):	Limited reliability in the expected value of the effect
D (weak)	Almost not reliable for the expected value of the effect

Strength of body of the evidence

### First-line treatment for unresectable advanced/recurrent gastric cancer

Since trastuzumab-containing regimens became the standard of care for HER2-positive gastric cancer, HER2 testing is strongly recommended in all patients who will receive chemotherapy for unresectable/metastatic gastric cancer. The methods of HER2 testing include immunohistochemistry and in situ hybridization (ISH).

#### HER2-negative gastric cancer

A combination of S-1 and cisplatin (SP) is the standard of care (the recommended regimen) based on the results of two phase III trials conducted in Japan (JCOG 9912 trial [41] and SPIRITS trial [42]) (evidence level A). After its non-inferiority to the 5-FU/cisplatin combination (FP) had been proven, a combination of capecitabine and cisplatin (XP) became one of standard treatments overseas and was employed as a control group in several global phase III studies including the ToGA trial [37] and AVAGAST trial [43]. Since safety and efficacy of XP have been recognized in subset analyses of the Japanese participants in these trials, this combination was added to the list of “Recommended regimens” (Evidence level A). CapeOX, a combination of capecitabine and oxaliplatin which was approved in 2014 in Japan, has shown efficacy that is at least equivalent to the FP in the subset analysis of a phase III study (no Japanese patients included) which evaluated triplets that combined these regimens with epirubicin (Evidence level B) [44]. A combination of S-1 and oxaliplatin (SOX) also demonstrated efficacy similar to SP in the G-SOX study (Evidence level B) [45]. These oxaliplatin-containing regimens could be delivered more easily than SP or XP because hydration is not required. Furthermore, a combination of 5-FU/levofolinate calcium (LV) with oxaliplatin (FOLFOX) has been employed as a control regimen in the recent comparative studies (Evidence level B) [46, 47] and has now been approved in Japan. FOLFOX can be particularly useful among patients who have difficulty in oral intake, such as those with bowel obstructions. To summarize, the list of “Recommended regimens” for the first-line treatment of unresectable advanced or recurrent gastric cancer includes various combinations of fluoropyrimidine and platinum except for the original FP regimen (Fig. 8), and selecting the most suitable regimen for each patient after taking into consideration various factors is a challenge for the physicians (CQ13).

A combination of S-1 and docetaxel failed to show superiority to S-1 monotherapy in the primary survival analysis of the START trial, but superiority in overall survival was observed in a reanalysis [48]. This regimen could be recommended for a limited population such as those who are not suitable to receive a platinum-containing regimen (“Conditionally recommended regimen”) (CQ14).

The combinations of irinotecan with cisplatin or S-1 are not recommended in the first-line treatment because they did not show significant superiority over S-1 alone in randomized trials conducted in Japan [41, 49].

Regarding the triplet regimens, superiority of adding docetaxel to a combination of infusional 5FU and cisplatin was proven in the V325 study [50] conducted in the Western countries. This triplet was avoided in Japan at the time, however, due to the excessive toxicity that did not balance well with the benefit in efficacy. More recently, following a promising phase II evidence, a triplet regimen consisting of S-1, cisplatin and docetaxel (DCS) was compared with SP in a phase III trial, JCOG1013. Since no benefit in overall survival was shown in that trial [51], triplet regimen containing taxane is currently not recommended as a first-line therapy.

Evidence is lacking regarding chemotherapy for specific types of patients such as those with impaired oral intake, peritoneal carcinomatosis (patients with a moderate to high volume of ascites or bowel obstruction) and the elderly. For these patients, conditionally recommended regimens could substitute for the recommended regimens as can be seen in CQ21.

#### HER2-positive gastric cancer

The definition of HER2 positive in the ToGA trial had been either IHC3+ or FISH positive [37]. In the subgroup analyses of the trial, survival benefit was more distinct among the IHC3+ or FISH positive/IHC2+ cohorts. Thus, trastuzumab-containing regimens are currently recommended for patients with IHC3+ or FISH positive/IHC2+ status in clinical practice. Since continuous infusion of 5-FU is rarely used nowadays, a combination of trastuzumab with XP which was employed in the ToGA study (Evidence level A), and combinations of trastuzumab with either triweekly or conventional SP where efficacy of both regimens were satisfactory in two successive phase II studies (Evidence level B) are the recommended regimens [52, 53].

In addition, results of phase II studies assessing combinations of trastuzumab with CapeOX [54] and SOX [55] have been reported. These regimens are rated as “Conditionally recommended regimens”, suitable for those who may not be able to tolerate cisplatin.

### Second-line treatment for unresectable advanced/recurrent gastric cancer

Second-line treatment is recommended for patients with sufficient performance status, because several randomized trials demonstrated significant survival benefit of chemotherapy over best supportive care (BSC), and favorable outcome was observed in a phase III trial that compared two chemotherapeutic regimens in the second-line setting.

Randomized trials conducted in Germany [56], Korea [57] and United Kingdom [58] revealed significant survival advantage of second-line chemotherapy (docetaxel or irinotecan) over BSC. A Japanese phase III trial, WJOG4007, failed to prove superiority in overall survival of irinotecan over paclitaxel (weekly administration), but the median survival time was approximately 9 months in both treatment groups: a favorable outcome when compared with survival data from other trials exploring the second-line chemotherapy [59]. Single-agent regimens with either docetaxel, irinotecan or paclitaxel (weekly administration), explored in the aforementioned trials, can now be selected as “Conditionally recommended regimens” when the paclitaxel/ramucirumab combination described below is considered unsuitable.

Since the paclitaxel/ramucirumab combination was shown to be superior to weekly paclitaxel monotherapy in a phase III trial (RAINBOW trial) [60], this regimen is currently the sole “Recommended regimen” (Evidence level A) (CQ16). In addition, the REGARD trial showed survival benefit of ramucirumab monotherapy over BSC. Thus, a monotherapy employing any of the agents including paclitaxel, docetaxel, irinotecan and ramucirumab, is a “Conditionally recommended regimen” when the paclitaxel/ramucirumab combination is deemed unsuitable. Nab-paclitaxel (albumin-conjugated paclitaxel) was approved in Japan in 2013. The clinical trial (ABSOLUTE trial) demonstrated non-inferiority of weekly administration of nab-paclitaxel over weekly paclitaxel monotherapy [61], and this regimen is also among the “Conditionally recommended regimens” when ramucirumab is not suitable. A combination of nab-paclitaxel and ramucirumab has also been established and could be used as a “Conditionally recommended regimen” when nab-paclitaxel is preferred over paclitaxel from, for example, the viewpoint of adverse reactions.

Efficacy of continuing trastuzumab beyond progression for HER2-positive gastric cancer initially treated with a trastuzumab-containing regimen has been denied by a randomized trial (CQ17).

## Adjuvant chemotherapy (CQ23–26)

### Clinical significance of postoperative adjuvant chemotherapy

Postoperative adjuvant chemotherapy is delivered with an intention to reduce recurrence by controlling residual tumor cells following a curative resection. Various regimens had been tested in numerous clinical trials in Japan without producing solid evidence in support of adjuvant chemotherapy until the efficacy of S-1 was proven in 2006 by the ACTS-GC trial [62, 63], a study that secured the place of postoperative S-1 monotherapy as a standard of care (Evidence level A). After this, the feasibility of several combinations of anticancer drug with S-1 was explored in the postoperative setting [64], of which a combination of S-1 and docetaxel was shown to have a significant benefit in relapse-free survival over S-1 alone in the interim analysis of a phase III trial JACCRO GC-07 for Stage III gastric cancer [65].

On the other hand, efficacy of the capecitabine + oxaliplatin combination in terms of relapse-free survival was demonstrated in 2002 in a phase III clinical trial conducted in Korea (CLASSIC) for TNM Stage II/III gastric cancer. [66] Subsequently, oxaliplatin in the postoperative adjuvant setting was approved for gastric cancer in Japan in November 2015 (Evidence level A). With bodies of evidence that appropriate postoperative adjuvant chemotherapy improves survival after curative resection, efforts to deliver the treatment at a preplanned dose and schedule while maintaining the general condition and managing the toxicities are now an essential component of treatment for Stage II/III gastric cancer.

### Indications

The patients eligible for the ACTS-GC trial were those with a tumor of pathological stage II, IIIA or IIIB, excluding those classified as stage II due to pT1, as defined by the previous 13th edition of the Japanese Classification of Gastric Carcinoma (2nd English edition), who had undergone R0 gastrectomy with ≥ D2 lymphadenectomy. The eligibility for postoperative adjuvant chemotherapy remains the same in the current treatment guidelines (CQ23). Note, however, that the stage grouping have been revised in the current 14th edition of the Japanese Classification of Gastric Carcinoma, and patients belonging to each pathological stage are not exactly the same as those at the time of the ATCS-GC trial.

### Palliative care

Palliative care is an approach that improves the quality of life of patients and their families facing the problems associated with life-threatening illness through the prevention and relief of suffering by means of early identification and impeccable assessment and treatment of pain and other problems, physical, psychosocial and spiritual (WHO Definition of Palliative Care, 2002). The importance of palliative care increases incrementally as cancer progresses. The knowledge and technique to cope with pain, to communicate and to manage symptoms are required. Methods to accomplish these aims include radiotherapy and psychotherapy in addition to medication. Various clinical studies are on-going with particular emphasis on pain control.

### Clinical pathway after surgery for gastric cancer

It is extremely difficult to establish a clinical pathway for patients undergoing gastric cancer surgery that is widely applicable to various surgical procedures. However, proposal of “a basic pathway” could contribute to reducing disparities in surgical management for gastric cancer among each institution. A basic pathway after surgery has been constructed around the timing of some core items such as removal of the nasogastric tube, initiation of oral fluid intake, initiation of solid food intake, administration of antibiotics, stoppage of intravenous fluid administration and discharge from the hospital (eight to 14 days after surgery) (Table 5). Criteria of discharge from the hospital include body temperature lower than 37 °C, the oral food intake more than one-third of normal condition and good control of the pain. This clinical pathway is applicable to all surgical procedures including total, distal and proximal gastrectomy regardless of whether the surgery was performed laparoscopically or by open approach. However, postoperative management should be individualized for high-risk patients with severe comorbidities that include impaired cardiac, pulmonary, hepatic or renal functions. Recently, investigators have been inclined to aim for further shortening of postoperative hospital stays through the concept of ERAS (enhanced recovery after surgery), but the value of such programs in gastric cancer surgery is yet to be defined.

**Table 5:** A common clinical pathway for distal, total and proximal gastrectomy

Clinical items	Date on the clinical pathway
Removal of nasogastric tube	Before or on postoperative day 1
Initiation of oral fluid intake	On or after postoperative day 1
Initiation of solid food intake	Between postoperative days 2–4
Prophylactic administration of antibiotics	Only on the day of operation
Removal of epidural tube	Before or on postoperative day 3
Removal of urinary catheter	Before or on postoperative day 3
Intravenous fluid administration	Until postoperative days 5–7
Removal of intra-abdominal drains	Before or on postoperative day 5
Discharge from the hospital	Between postoperative days 8–14

### Follow-up surveillance after surgery for gastric cancer

**Fig. 10:**
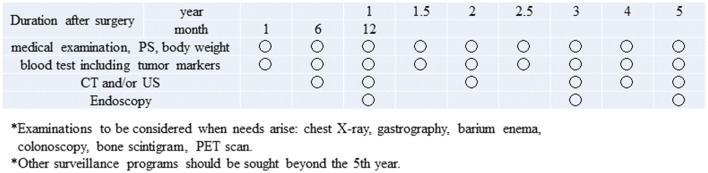
Postoperative follow-up for Stage I gastric cancer patients

**Fig. 11:**
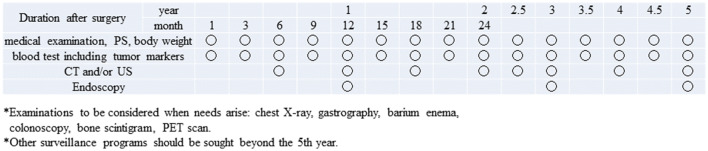
Postoperative follow-up for Stage II–III gastric cancer patients

Follow-up at the outpatient clinic could be helpful, so that the patients can readjust to their lives at home, cope with postgastrectomy symptoms and overcome the nutritional issues. In addition, surveillance for early detection of recurrence and secondary cancer is usually conducted according to the level of risk for recurrence, estimated based on the clinical stages. However, evidence that such surveillance actually improves survival is lacking. Due to the paucity of prospective studies that explored follow-up programs after gastrectomy, it is not possible to make any recommendation on how often the examinations should be performed, or even on which examination to perform. However, some retrospective studies suggest that CT, measurement of tumor markers (CEA and CA19-9) and endoscopy are effective to detect recurrence, gastric remnant cancer and metachronous multiple cancer. Tumor markers, when appropriate, are apt to rise 2–3 months before metastatic lesions become detectable by imaging modalities. Models of follow-up programs for early-stage cancer and R0 resected advanced cancer are shown in Figs. 10 and 11.

Follow-up should continue for no longer than 5 years after which patients should be referred to regional general physicians or should be encouraged to undergo surveillance examinations provided as a part of health care programs in their districts or at their places of work. In that aspect, collaboration among various levels of medical facilities is needed to provide comprehensive care to gastric cancer survivors. Ultimately, there remains a need to scientifically verify the prognostic relevance of postoperative follow-up programs.

### Clinical questions for surgery

*CQ1* Is gastrectomy as reduction surgery for advanced gastric cancer with incurable factors recommended for improvement of prognosis?

*Recommendation* It is strongly recommended not to perform gastrectomy as reduction surgery.

*CQ2.* Is Pylorus-preserving gastrectomy (PPG) recommended for early gastric cancer?

*Recommendation* PPG for early gastric cancer in the middle portion of the stomach is weakly recommended.

*CQ3* Is proximal gastrectomy recommended for cT1N0 tumor in the upper-third stomach when EMR or ESD is not indicated?

*Recommendation* Proximal gastrectomy is weakly recommended as an option for cT1N0 tumor in the upper-third stomach.

*CQ4* Is prophylactic splenectomy to dissect Nos. 10 and 11 lymph nodes recommended in advanced gastric cancer of the upper-third stomach?

*Recommendation* It is strongly recommended not to perform splenectomy for advanced gastric cancer in the upper-third stomach which does not invade the greater curvature.

*CQ5* Is extended gastrectomy with neoadjuvant chemotherapy recommended in patients with extensive lymph node metastases which are borderline resectable?

*Recommendation* Extended gastrectomy with neoadjuvant chemotherapy is weakly recommended for cases that fulfill the following criteria and have no other non-curative factors: a small number of nodal swelling limited to the No.16a2, b1 region and/or swollen lymph nodes that are borderline resectable around the branches of celiac artery (see CQ26).

*CQ6* What is the optimal extent of lymphadenectomy for esophagogastric junctional cancer?

*Recommendation* Lymph node station Nos. 1, 2, 3, 7, and lower mediastinal lymph nodes which can be resected in proximal gastrectomy and lower esophageal resection form the basis of systematic lymphadenectomy for this cohort. Subtotal esophagectomy with lymph node dissection of the superior and middle mediastinum could be considered, depending on the histologic type, diameter of the tumor, distance from the esophagogastric junction to the oral edge of the tumor (refer to Fig. 6).

*CQ7* Is laparoscopic total gastrectomy for tumor in the upper-third stomach recommended?

*Recommendation* Laparoscopic total gastrectomy for tumor in the upper-third stomach can be considered for cStage I tumor. However, the body of evidence to support this procedure remains insufficient. This procedure should be conducted by a team centered around a well-experienced laparoscopic surgeon.

*CQ8* Is hepatectomy recommended for metastasis from gastric cancer?

*Recommendation* Surgical resection is weakly recommended for cases with small number of metastases with no other incurable factor.

*CQ9* What is the optimal extent of lymphadenectomy for cancer of the gastric remnant ≥ cT2?

*Recommendation* Lymphadenectomy of the regional lymph nodes of the stomach which had not been resected at the initial surgery is recommended. Clinical benefit of dissecting meso-jejunal lymph nodes and splenic hilar lymph nodes (No. 10) has not been established.

*CQ10* Is staging laparoscopy recommended to decide the treatment plan for gastric cancer?

*Recommendation* Staging laparoscopy is weakly recommended to decide on the treatment plan for patients with relatively high risk of peritoneal dissemination, referring also to the results of peritoneal lavage cytology using samples that are collected at staging laparoscopy. This procedure is particularly useful for advanced gastric cancer patients who can be indicated for neoadjuvant chemotherapy.

### Clinical questions for endoscopic resection

*CQ11* Which method of endoscopic resection (EMR or ESD) is recommended for a lesion of EMR/ESD absolute indication (differentiated-type adenocarcinoma, UL0, T1a, diameter ≤ 2 cm)?

*Recommendation* ESD is weakly recommended for a lesion classified as EMR/ESD absolute indication (differentiated-type adenocarcinoma, UL0, T1a, diameter ≤ 2 cm).

*CQ12* Is *Helicobacter pylori* eradication after endoscopic resection recommended for a *Helicobacter pylori*-positive gastric cancer patient?

*Recommendation Helicobacter pylori* eradication after endoscopic resection is weakly recommended for a *Helicobacter pylori*-positive gastric cancer patient.

### Clinical questions for chemotherapy

#### Clinical question regarding chemotherapy for unresectable advanced/recurrent gastric cancer (AGC)

*CQ13* Should an appropriate fluoropyrimidine/platinum combination for the first-line treatment of AGC be selected based on the route of administration and toxicity profile?

*Recommendation* In the first-line treatment of AGC, it is weakly recommended to select an appropriate fluoropyrimidine/platinum combination from numerous options based on the route of administration and toxicity profile.

*CQ14* Are the taxanes recommended for the first-line treatment of AGC?

*Recommendations* Taxanes are conditionally recommended for the first-line treatment of AGC when platinum is considered unsuitable.

*CQ15* Is the continued use of fluoropyrimidine alone in the first-line treatment after termination of the platinum due to reasons other than disease progression recommended in the treatment of AGC?

*Recommendation* Continuation of fluoropyrimidine alone until disease progression is strongly recommended after termination of the platinum due to reasons other than disease progression.

*CQ16* Is a monotherapy recommended for the second-line treatment of AGC?

*Recommendation* Monotherapy for second-line treatment of AGC is conditionally recommended.

*CQ17* Is administration of trastuzumab beyond progression recommended in the second-line treatment of HER2-positive AGC?

*Recommendation* It is recommended not to administer trastuzumab beyond progression is not recommended in the second-line treatment of HER2-positive AGC.

*CQ18* Is administration of S-1 beyond progression in the second-line treatment of AGC recommended?

*Recommendation* It is recommended not to administer S-1 beyond progression in the second-line treatment of AGC.

*CQ19* Is chemotherapy recommended as third- or later-line treatment of AGC?

*Recommendation* Nivolumab or irinotecan monotherapy is recommended for third- or later-line treatment of AGC.

*CQ20* Is chemotherapy recommended for patients with peritoneal lavage cytology-positive (CY1) status after gastrectomy?

*Recommendation* Chemotherapy is recommended for patients with peritoneal lavage cytology-positive (CY1) status who underwent gastrectomy.

*CQ21* Is chemotherapy recommended for patients with impaired oral intake or massive ascites due to extensive peritoneal disease?

*Recommendation* Chemotherapy is conditionally recommended for patients with impaired oral intake or massive ascites after discreet assessment of general condition.

*CQ22* Is chemotherapy recommended for an elderly patient with AGC?

*Recommendation* A chemotherapy is conditionally recommended for an elderly patient, based on a discreet assessment of the general condition and appropriate selection of the treatment regimen.

#### Clinical questions for perioperative chemotherapy

*CQ23.* Is it recommended to select adjuvant chemotherapy regimen based on pathological stage or histological type of tumor?

*Recommendation* S-1 monotherapy is recommended for an adjuvant chemotherapy of stage II gastric cancer. S-1 monotherapy or an oxaliplatin-based combination such as CapeOX is recommended for an adjuvant chemotherapy for stage III gastric cancer after considering risk and benefit for each patient.

*CQ24* Should a patient who had recurrence during or within 6 months after termination of the adjuvant chemotherapy be re-challenged with the drugs which had been used in the adjuvant chemotherapy?

*Recommendation* It is recommended not to use chemotherapy consisting of the drugs which had already been administered in the adjuvant setting to treat cancer that recurred during or within 6 months after termination of the adjuvant chemotherapy.

*CQ25* Is postoperative adjuvant chemotherapy recommended for Stage IV gastric cancer after R0 resection?

*Recommendation* Postoperative adjuvant chemotherapy is recommended for Stage IV gastric cancer after R0 resection.

*CQ26* Is neoadjuvant chemotherapy recommended for resectable advanced gastric cancer?

*Recommendations* Neoadjuvant chemotherapy is conditionally recommended for a patient with extensive lymph node metastasis (see also CQ5).
